# [1,5-Bis(2-meth­oxy­phen­yl)thio­carbazo­nato-κ^2^ *N* ^1^,*S*]phenyl­mercury(II)

**DOI:** 10.1107/S1600536811049099

**Published:** 2011-11-23

**Authors:** Karel von Eschwege, Fabian Muller, Alfred Muller

**Affiliations:** a Department of Chemistry, University of the Free State, PO Box 339, Bloemfontein 9300, South Africa; b Research Center for Synthesis and Catalysis, Department of Chemistry, University of Johannesburg (APK Campus), PO Box 524, Auckland Park, Johannesburg 2006, South Africa

## Abstract

The title compound, [Hg(C_6_H_5_)(C_15_H_15_N_4_O_2_S)], shows the metal–phenyl moiety coordinated out of plane with the thio­carbazo­nate ligand by 43.84 (6)°. Important geometrical parameters include Hg—S = 2.3653 (10) Å, Hg—C = 2.058 (4) Å and S—Hg—C = 179.06 (11)°. There is a weak coordination of an N atom of the ligand to Hg [Hg—N = 2.725 (3) Å]. S⋯Hg inter­actions[3.2928 (10) Å] form chains along [001], stabilizing the crystal structure.

## Related literature

For general background to thio­carbazo­natomercury(II) complexes, see: Irving *et al.* (1949 ▶); Webb *et al.* (1950 ▶); Hutton *et al.* (1980 ▶); Von Eschwege *et al.* (2011 ▶); Schwoerer *et al.* (2011 ▶). For synthetic procedures relating to the title compound, see: Mirkhalaf *et al.* (1998 ▶); Von Eschwege *et al.* (2008 ▶).
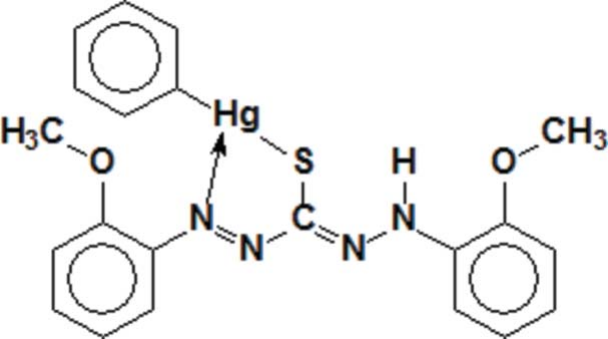


## Experimental

### 

#### Crystal data

[Hg(C_6_H_5_)(C_15_H_15_N_4_O_2_S)]
*M* *_r_* = 593.06
Monoclinic, 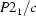

*a* = 15.2113 (16) Å
*b* = 18.2730 (18) Å
*c* = 7.4649 (8) Å
β = 90.106 (2)°
*V* = 2074.9 (4) Å^3^
*Z* = 4
Mo *K*α radiation
μ = 7.54 mm^−1^
*T* = 299 K
0.26 × 0.19 × 0.01 mm

#### Data collection

Bruker APEX DUO 4K CCD diffractometer
Absorption correction: multi-scan (*SADABS*; Bruker, 2008 ▶) *T*_min_ = 0.542, *T*_max_ = 0.746
17400 measured reflections
5107 independent reflections
4107 reflections with *I* > 2σ(*I*)
*R*_int_ = 0.042

#### Refinement

*R*[*F* ^2^ > 2σ(*F* ^2^)] = 0.030
*wR*(*F* ^2^) = 0.076
*S* = 1.03
5107 reflections
264 parameters
H-atom parameters constrained
Δρ_max_ = 1.16 e Å^−3^
Δρ_min_ = −0.89 e Å^−3^

### 

Data collection: *APEX2* (Bruker, 2011 ▶); cell refinement: *SAINT* (Bruker, 2008 ▶); data reduction: *SAINT* and *XPREP* (Bruker, 2008 ▶); program(s) used to solve structure: *SIR97* (Altomare *et al.*, 1999 ▶); program(s) used to refine structure: *SHELXL97* (Sheldrick, 2008 ▶); molecular graphics: *DIAMOND* (Brandenburg & Putz, 2005 ▶); software used to prepare material for publication: *WinGX* (Farrugia, 1999 ▶).

## Supplementary Material

Crystal structure: contains datablock(s) global, I. DOI: 10.1107/S1600536811049099/zq2138sup1.cif

Structure factors: contains datablock(s) I. DOI: 10.1107/S1600536811049099/zq2138Isup2.hkl

Additional supplementary materials:  crystallographic information; 3D view; checkCIF report

## Figures and Tables

**Fig. 1.**
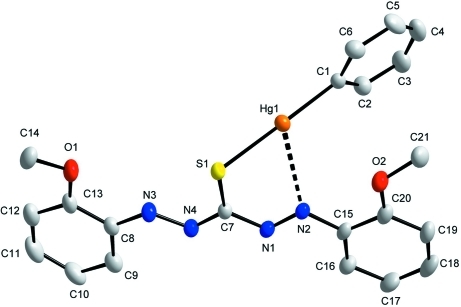
View of the title compound indicating labeling and displacement ellipsoids (drawn at a 50% probability level).

**Fig. 2.**
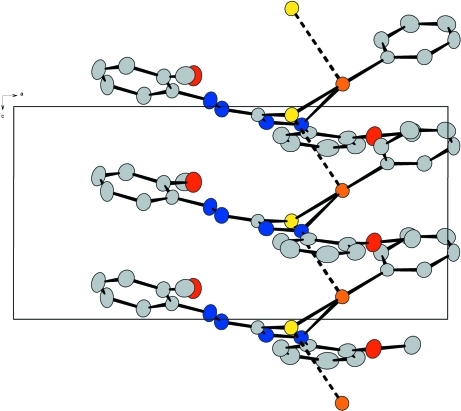
Partial packing diagram of the title compound viewed along the b axis illustrating the Hg···S interactions stabilizing the crystal packing.

## supplementary crystallographic information

### Comment

Irving *et al.* (1949) and Webb *et al.* (1950) independently reported photochromicity of the thiocarbazonatomercury(II) complex. The single-crystal X-ray structure of the phenyl mercury thiocarbazonate complex was established by Hutton *et al.* (1980) and redox properties by Von Eschwege *et al.* (2011), while femtosecond laser spectroscopy resolved the short-lived time constants of the photochromic reaction (Schwoerer *et al.*, 2011). For the purpose of investigating the influence of electron donating groups on the photochromic and redox reactions of thiocarbazonatophenylmercury(II) complexes a series of electronically altered dithizones were synthesized and for the first time complexed with mercury. Deep orange-red needle crystals of the *ortho*-methoxy derivative, suitable for X-ray crystallography, were isolated from a dichloromethane solution overlaid with ethanol.

The title compound (Fig. 1, Table 1) shows the metal-phenyl moiety coordinated out of plane to the (2-methoxyphenyl)thiocarbazonate by 43.84 (6)°. The methoxy moieties are slightly twisted out of planarity with their respective phenyl rings [C12—C13—O1—C14 = 22.0 (7)° and C19—C20—O2—C21 = 16.1 (6)°]. Important geometrical parameters include Hg—S = 2.3653 (10) Å, Hg—C = 2.058 (4) Å, and 〈 S—Hg—C = 179.06 (11)°. There is a weak coordination of a N-atom of the thiocarbazonate to Hg (Hg—N = 2.725 (3) Å). S···Hg interactions stabilizes the crystal packing (Fig. 2).

### Experimental

Solvents (AR) purchased from Merck and reagents from Sigma-Aldrich were used without further purification. The *ortho*-methoxy derivative of dithizone, (*o*-OCH_3_PhNHN)_2_CS, was prepared according to a procedure reported by Mirkhalaf *et al.* (1998). The synthesis and crystallization of the title compound was done according to a procedure earlier reported by Von Eschwege *et al.* (2008).

### Refinement

All hydrogen atoms were positioned in geometrically idealized positions with C—H = 0.98 Å (methyl), 0.95 Å (aromatic) and 0.86 Å (imine). All hydrogen atoms were allowed to ride on their parent atoms with *U*_iso_(H) = 1.2*U*_eq_(C), except for the methyl where *U*_iso_(H) = 1.5*U*_eq_(C) was utilized. The initial positions of methyl hydrogen atoms were located from a Fourier difference map and refined as fixed rotor. The highest residual electron density of 1.15 e.Å^-3^ is 0.81 Å from Hg1 representing no physical meaning.

### Crystal data

**Table:**

[Hg(C_6_H_5_)(C_15_H_15_N_4_O_2_S)]	*F*(000) = 1144
*M**_r_* = 593.06	*D*_x_ = 1.895 Mg m^−^^3^
Monoclinic, *P*2_1_/*c*	Mo *K*α radiation, λ = 0.71073 Å
Hall symbol: -P 2ybc	Cell parameters from 5470 reflections
*a* = 15.2113 (16) Å	θ = 2.7–25.7°
*b* = 18.2730 (18) Å	µ = 7.54 mm^−^^1^
*c* = 7.4649 (8) Å	*T* = 299 K
β = 90.106 (2)°	Plate, brown
*V* = 2074.9 (4) Å^3^	0.26 × 0.19 × 0.01 mm
*Z* = 4	

### Data collection

**Table:**

Bruker APEX DUO 4K CCD diffractometer	5107 independent reflections
graphite	4107 reflections with *I* > 2σ(*I*)
Detector resolution: 8.4 pixels mm^-1^	*R*_int_ = 0.042
φ and ω scans	θ_max_ = 28.3°, θ_min_ = 1.3°
Absorption correction: multi-scan (*SADABS*; Bruker, 2008)	*h* = −20→19
*T*_min_ = 0.542, *T*_max_ = 0.746	*k* = −24→24
17400 measured reflections	*l* = −9→9

### Refinement

**Table:**

Refinement on *F*^2^	Primary atom site location: structure-invariant direct methods
Least-squares matrix: full	Secondary atom site location: difference Fourier map
*R*[*F*^2^ > 2σ(*F*^2^)] = 0.030	Hydrogen site location: inferred from neighbouring sites
*wR*(*F*^2^) = 0.076	H-atom parameters constrained
*S* = 1.03	*w* = 1/[σ^2^(*F*_o_^2^) + (0.036*P*)^2^] where *P* = (*F*_o_^2^ + 2*F*_c_^2^)/3
5107 reflections	(Δ/σ)_max_ = 0.001
264 parameters	Δρ_max_ = 1.16 e Å^−^^3^
0 restraints	Δρ_min_ = −0.89 e Å^−^^3^

### Special details

**Table:**

Experimental. The intensity data was collected on a Bruker Apex DUO 4 K CCD diffractometer using an exposure time of 60 s/frame. A total of 894 frames were collected with a frame width of 0.5° covering up to θ = 28.26° with 99.2% completeness accomplished.Analytical data: *M*.p. 212 - 213 °C; λ_max_ (dichloromethane) 505 nm; δ_H_ (300 MHz, CDCl_3_) 3.68, 4.03 (6 H, 2 × s, 2 × CH_3_), 6.57 – 7.89 (13 H, m, 2 × C_6_H_4_, 1 × C_6_H_5_), 9.75 (1*H*, s, 1 × NH).
Geometry. All e.s.d.'s (except the e.s.d. in the dihedral angle between two l.s. planes) are estimated using the full covariance matrix. The cell e.s.d.'s are taken into account individually in the estimation of e.s.d.'s in distances, angles and torsion angles; correlations between e.s.d.'s in cell parameters are only used when they are defined by crystal symmetry. An approximate (isotropic) treatment of cell e.s.d.'s is used for estimating e.s.d.'s involving l.s. planes.
Refinement. Refinement of *F*^2^ against ALL reflections. The weighted *R*-factor *wR* and goodness of fit *S* are based on *F*^2^, conventional *R*-factors *R* are based on *F*, with *F* set to zero for negative *F*^2^. The threshold expression of *F*^2^ > σ(*F*^2^) is used only for calculating *R*-factors(gt) *etc*. and is not relevant to the choice of reflections for refinement. *R*-factors based on *F*^2^ are statistically about twice as large as those based on *F*, and *R*- factors based on ALL data will be even larger.

### Fractional atomic coordinates and isotropic or equivalent isotropic displacement parameters (Å^2^)

**Table:**

	*x*	*y*	*z*	*U*_iso_*/*U*_eq_	
Hg1	0.757410 (11)	0.741409 (8)	0.89423 (2)	0.04159 (7)	
S1	0.63975 (7)	0.80179 (5)	1.03894 (13)	0.0396 (2)	
C1	0.8609 (3)	0.6905 (2)	0.7679 (5)	0.0400 (9)	
C2	0.8465 (3)	0.6229 (2)	0.6869 (6)	0.0517 (11)	
H2	0.7896	0.6043	0.6847	0.062*	
C3	0.9118 (4)	0.5830 (3)	0.6110 (7)	0.0672 (14)	
H3	0.8999	0.5377	0.5596	0.081*	
C4	0.9962 (4)	0.6106 (3)	0.6112 (6)	0.0723 (16)	
H4	1.0415	0.584	0.5587	0.087*	
C5	1.0137 (3)	0.6760 (3)	0.6869 (6)	0.0696 (15)	
H5	1.0707	0.6944	0.686	0.084*	
C6	0.9459 (3)	0.7164 (3)	0.7672 (6)	0.0560 (11)	
H6	0.9586	0.7612	0.8204	0.067*	
C7	0.5620 (3)	0.73001 (19)	1.0383 (5)	0.0373 (8)	
C8	0.3652 (3)	0.8205 (2)	0.9259 (5)	0.0431 (9)	
C9	0.2994 (3)	0.7691 (2)	0.9489 (7)	0.0542 (11)	
H9	0.3123	0.7235	0.998	0.065*	
C10	0.2150 (4)	0.7859 (3)	0.8986 (8)	0.0692 (14)	
H10	0.1706	0.7515	0.9132	0.083*	
C11	0.1954 (4)	0.8535 (3)	0.8264 (7)	0.0748 (16)	
H11	0.1383	0.8637	0.7897	0.09*	
C12	0.2595 (4)	0.9059 (3)	0.8082 (7)	0.0646 (13)	
H12	0.2457	0.9516	0.7611	0.078*	
C13	0.3446 (3)	0.8903 (2)	0.8604 (6)	0.0494 (10)	
C14	0.3947 (4)	1.0137 (2)	0.8541 (7)	0.0654 (14)	
H14A	0.3669	1.0265	0.7429	0.098*	
H14B	0.4483	1.041	0.867	0.098*	
H14C	0.356	1.0251	0.9517	0.098*	
C15	0.6791 (3)	0.5672 (2)	1.1234 (5)	0.0417 (9)	
C16	0.6159 (3)	0.5142 (2)	1.1353 (6)	0.0522 (11)	
H16	0.5572	0.5272	1.1201	0.063*	
C17	0.6369 (4)	0.4415 (2)	1.1693 (6)	0.0648 (14)	
H17	0.5931	0.406	1.1747	0.078*	
C18	0.7232 (4)	0.4233 (2)	1.1947 (7)	0.0683 (16)	
H18	0.7377	0.375	1.2202	0.082*	
C19	0.7891 (4)	0.4745 (2)	1.1834 (6)	0.0593 (13)	
H19	0.8474	0.4607	1.1998	0.071*	
C20	0.7683 (3)	0.5471 (2)	1.1474 (5)	0.0507 (11)	
C21	0.9188 (4)	0.5815 (3)	1.1217 (7)	0.0700 (15)	
H21A	0.9403	0.564	1.2348	0.105*	
H21B	0.9529	0.6231	1.0849	0.105*	
H21C	0.9239	0.5435	1.0336	0.105*	
N1	0.5817 (2)	0.65684 (16)	1.0733 (4)	0.0425 (8)	
N2	0.6628 (2)	0.64165 (16)	1.0849 (4)	0.0387 (7)	
N3	0.4523 (2)	0.80730 (17)	0.9711 (4)	0.0468 (8)	
H3A	0.4893	0.8429	0.9729	0.056*	
N4	0.4790 (3)	0.73963 (16)	1.0117 (5)	0.0447 (8)	
O1	0.4138 (2)	0.93781 (16)	0.8552 (5)	0.0618 (9)	
O2	0.8295 (2)	0.60215 (16)	1.1388 (4)	0.0585 (8)	

### Atomic displacement parameters (Å^2^)

**Table:**

	*U*^11^	*U*^22^	*U*^33^	*U*^12^	*U*^13^	*U*^23^
Hg1	0.03951 (11)	0.04266 (10)	0.04260 (10)	0.00361 (6)	0.00085 (7)	−0.00262 (6)
S1	0.0410 (6)	0.0322 (5)	0.0458 (5)	0.0036 (4)	−0.0026 (4)	−0.0019 (4)
C1	0.040 (2)	0.046 (2)	0.0335 (17)	0.0076 (17)	0.0009 (17)	0.0040 (15)
C2	0.048 (3)	0.048 (2)	0.059 (3)	0.001 (2)	0.001 (2)	−0.0059 (19)
C3	0.077 (4)	0.059 (3)	0.066 (3)	0.016 (3)	0.007 (3)	−0.011 (2)
C4	0.075 (4)	0.085 (4)	0.057 (3)	0.036 (3)	0.008 (3)	−0.002 (3)
C5	0.033 (3)	0.112 (5)	0.064 (3)	0.005 (3)	−0.002 (2)	0.002 (3)
C6	0.049 (3)	0.068 (3)	0.051 (2)	−0.004 (2)	−0.003 (2)	−0.002 (2)
C7	0.038 (2)	0.0358 (19)	0.0384 (19)	0.0047 (16)	−0.0027 (17)	0.0005 (14)
C8	0.037 (2)	0.048 (2)	0.045 (2)	0.0062 (18)	−0.0038 (18)	−0.0074 (16)
C9	0.046 (3)	0.053 (3)	0.064 (3)	0.002 (2)	−0.003 (2)	−0.013 (2)
C10	0.037 (3)	0.086 (4)	0.085 (4)	−0.003 (3)	0.001 (3)	−0.023 (3)
C11	0.042 (3)	0.098 (4)	0.084 (4)	0.019 (3)	−0.014 (3)	−0.023 (3)
C12	0.056 (3)	0.076 (3)	0.062 (3)	0.025 (3)	−0.005 (3)	−0.005 (3)
C13	0.043 (3)	0.052 (2)	0.053 (2)	0.015 (2)	−0.003 (2)	−0.0067 (19)
C14	0.083 (4)	0.045 (3)	0.068 (3)	0.014 (2)	−0.005 (3)	0.004 (2)
C15	0.059 (3)	0.0331 (19)	0.0331 (17)	0.0093 (18)	−0.0033 (18)	0.0028 (14)
C16	0.060 (3)	0.038 (2)	0.059 (2)	0.002 (2)	−0.004 (2)	0.0056 (18)
C17	0.085 (4)	0.037 (2)	0.072 (3)	−0.004 (2)	0.004 (3)	0.009 (2)
C18	0.113 (5)	0.031 (2)	0.060 (3)	0.013 (3)	−0.001 (3)	0.0060 (19)
C19	0.074 (4)	0.045 (2)	0.059 (3)	0.021 (2)	−0.008 (3)	0.004 (2)
C20	0.068 (3)	0.042 (2)	0.041 (2)	0.013 (2)	−0.007 (2)	0.0029 (16)
C21	0.059 (4)	0.081 (4)	0.070 (3)	0.017 (3)	−0.007 (3)	0.018 (3)
N1	0.050 (2)	0.0321 (16)	0.0458 (17)	0.0046 (15)	0.0015 (16)	0.0035 (13)
N2	0.044 (2)	0.0336 (16)	0.0382 (16)	0.0050 (14)	−0.0023 (15)	0.0028 (12)
N3	0.040 (2)	0.0373 (17)	0.063 (2)	0.0039 (15)	−0.0067 (17)	−0.0015 (15)
N4	0.043 (2)	0.0369 (17)	0.054 (2)	0.0047 (15)	−0.0005 (18)	0.0004 (14)
O1	0.058 (2)	0.0399 (16)	0.088 (2)	0.0125 (15)	−0.0015 (18)	0.0012 (15)
O2	0.053 (2)	0.0465 (17)	0.076 (2)	0.0043 (15)	−0.0084 (17)	0.0095 (15)

### Geometric parameters (Å, °)

**Table:**

Hg1—C1	2.058 (4)	C12—H12	0.93
Hg1—S1	2.3653 (10)	C13—O1	1.364 (5)
S1—C7	1.766 (4)	C14—O1	1.417 (5)
C1—C6	1.378 (6)	C14—H14A	0.96
C1—C2	1.392 (6)	C14—H14B	0.96
C2—C3	1.358 (6)	C14—H14C	0.96
C2—H2	0.93	C15—C16	1.368 (6)
C3—C4	1.380 (8)	C15—N2	1.412 (4)
C3—H3	0.93	C15—C20	1.417 (6)
C4—C5	1.348 (7)	C16—C17	1.389 (6)
C4—H4	0.93	C16—H16	0.93
C5—C6	1.403 (7)	C17—C18	1.368 (7)
C5—H5	0.93	C17—H17	0.93
C6—H6	0.93	C18—C19	1.374 (7)
C7—N4	1.289 (6)	C18—H18	0.93
C7—N1	1.395 (4)	C19—C20	1.389 (5)
C8—C9	1.384 (6)	C19—H19	0.93
C8—N3	1.387 (5)	C20—O2	1.372 (5)
C8—C13	1.401 (6)	C21—O2	1.416 (6)
C9—C10	1.372 (8)	C21—H21A	0.96
C9—H9	0.93	C21—H21B	0.96
C10—C11	1.380 (8)	C21—H21C	0.96
C10—H10	0.93	N1—N2	1.267 (5)
C11—C12	1.373 (8)	N3—N4	1.336 (4)
C11—H11	0.93	N3—H3A	0.86
C12—C13	1.381 (7)		
			
C1—Hg1—S1	179.06 (11)	C12—C13—C8	119.7 (5)
C7—S1—Hg1	99.19 (13)	O1—C14—H14A	109.5
C6—C1—C2	116.7 (4)	O1—C14—H14B	109.5
C6—C1—Hg1	124.4 (3)	H14A—C14—H14B	109.5
C2—C1—Hg1	118.7 (3)	O1—C14—H14C	109.5
C3—C2—C1	123.0 (5)	H14A—C14—H14C	109.5
C3—C2—H2	118.5	H14B—C14—H14C	109.5
C1—C2—H2	118.5	C16—C15—N2	124.9 (4)
C2—C3—C4	118.9 (5)	C16—C15—C20	118.7 (4)
C2—C3—H3	120.5	N2—C15—C20	116.3 (4)
C4—C3—H3	120.5	C15—C16—C17	121.8 (5)
C5—C4—C3	120.5 (5)	C15—C16—H16	119.1
C5—C4—H4	119.8	C17—C16—H16	119.1
C3—C4—H4	119.8	C18—C17—C16	118.6 (5)
C4—C5—C6	120.1 (5)	C18—C17—H17	120.7
C4—C5—H5	119.9	C16—C17—H17	120.7
C6—C5—H5	119.9	C17—C18—C19	121.7 (4)
C1—C6—C5	120.7 (5)	C17—C18—H18	119.1
C1—C6—H6	119.6	C19—C18—H18	119.1
C5—C6—H6	119.6	C18—C19—C20	119.8 (5)
N4—C7—N1	111.7 (4)	C18—C19—H19	120.1
N4—C7—S1	123.7 (3)	C20—C19—H19	120.1
N1—C7—S1	124.5 (3)	O2—C20—C19	123.7 (5)
C9—C8—N3	122.9 (4)	O2—C20—C15	116.9 (3)
C9—C8—C13	120.0 (4)	C19—C20—C15	119.3 (5)
N3—C8—C13	117.1 (4)	O2—C21—H21A	109.5
C10—C9—C8	119.4 (5)	O2—C21—H21B	109.5
C10—C9—H9	120.3	H21A—C21—H21B	109.5
C8—C9—H9	120.3	O2—C21—H21C	109.5
C9—C10—C11	120.6 (5)	H21A—C21—H21C	109.5
C9—C10—H10	119.7	H21B—C21—H21C	109.5
C11—C10—H10	119.7	N2—N1—C7	115.6 (3)
C12—C11—C10	120.6 (5)	N1—N2—C15	113.3 (3)
C12—C11—H11	119.7	N4—N3—C8	120.4 (3)
C10—C11—H11	119.7	N4—N3—H3A	119.8
C11—C12—C13	119.6 (5)	C8—N3—H3A	119.8
C11—C12—H12	120.2	C7—N4—N3	117.3 (4)
C13—C12—H12	120.2	C13—O1—C14	117.7 (4)
O1—C13—C12	125.7 (4)	C20—O2—C21	117.4 (4)
O1—C13—C8	114.7 (4)		
			
C6—C1—C2—C3	0.5 (7)	C15—C16—C17—C18	1.2 (7)
Hg1—C1—C2—C3	−175.5 (4)	C16—C17—C18—C19	−1.4 (8)
C1—C2—C3—C4	−1.0 (8)	C17—C18—C19—C20	0.8 (8)
C2—C3—C4—C5	0.5 (8)	C18—C19—C20—O2	178.1 (4)
C3—C4—C5—C6	0.3 (8)	C18—C19—C20—C15	0.2 (6)
C2—C1—C6—C5	0.4 (6)	C16—C15—C20—O2	−178.4 (4)
Hg1—C1—C6—C5	176.1 (3)	N2—C15—C20—O2	2.8 (5)
C4—C5—C6—C1	−0.8 (7)	C16—C15—C20—C19	−0.4 (6)
Hg1—S1—C7—N4	−141.9 (3)	N2—C15—C20—C19	−179.2 (4)
Hg1—S1—C7—N1	40.9 (3)	N4—C7—N1—N2	173.9 (3)
N3—C8—C9—C10	−179.1 (4)	S1—C7—N1—N2	−8.6 (5)
C13—C8—C9—C10	3.2 (7)	C7—N1—N2—C15	179.0 (3)
C8—C9—C10—C11	−0.3 (8)	C16—C15—N2—N1	4.5 (5)
C9—C10—C11—C12	−1.9 (8)	C20—C15—N2—N1	−176.8 (3)
C10—C11—C12—C13	1.0 (8)	C9—C8—N3—N4	11.1 (6)
C11—C12—C13—O1	−177.8 (4)	C13—C8—N3—N4	−171.1 (4)
C11—C12—C13—C8	2.0 (7)	N1—C7—N4—N3	−177.9 (3)
C9—C8—C13—O1	175.7 (4)	S1—C7—N4—N3	4.5 (5)
N3—C8—C13—O1	−2.1 (5)	C8—N3—N4—C7	174.5 (4)
C9—C8—C13—C12	−4.1 (6)	C12—C13—O1—C14	22.0 (7)
N3—C8—C13—C12	178.1 (4)	C8—C13—O1—C14	−157.7 (4)
N2—C15—C16—C17	178.4 (4)	C19—C20—O2—C21	16.1 (6)
C20—C15—C16—C17	−0.3 (6)	C15—C20—O2—C21	−166.0 (4)
